# Prevalence of Mastitis Pathogens and Antimicrobial Susceptibility of Isolates From Cattle and Buffaloes in Northwest of Pakistan

**DOI:** 10.3389/fvets.2021.746755

**Published:** 2021-10-14

**Authors:** Tariq Ali, Kamran, Abdur Raziq, Inamullah Wazir, Rafi Ullah, Pervaiz Shah, Muhammad Ijaz Ali, Bo Han, Gang Liu

**Affiliations:** ^1^College of Veterinary Medicine, China Agricultural University, Beijing, China; ^2^Center of Microbiology and Biotechnology, Veterinary Research Institute, Peshawar, Pakistan; ^3^College of Veterinary Sciences, University of Agriculture, Peshawar, Pakistan

**Keywords:** bovine mastitis, bacteriology, antimicrobial susceptibility, cattle, buffaloes

## Abstract

Mastitis is the most prevalent disease of dairy animals, imparting huge economic losses to the dairy industry. There is always a dire need to monitor the prevalence of mastitis, its bacteriology, and evaluation of antimicrobial susceptibilities for mastitis control and prevention. Therefore, the objectives of this study were to investigate: (i) the prevalence of mastitis in cattle and buffaloes; (ii) identification of bacteria associated with mastitis; (iii) antimicrobial susceptibility of bacterial isolates. Milk samples (*n* = 1,566) from cattle (*n* = 1,096) and buffaloes (*n* = 470) were processed for detection of mastitis using the California mastitis test in the year 2018–19. A total of 633 mastitic milk samples were further processed for bacteriology and antimicrobial susceptibility testing by the disc diffusion method. Overall, the prevalence of clinical and subclinical mastitis was 17 and 57% in both species. Clinical mastitis was higher in cattle (20%) compared to buffaloes (11%), whereas subclinical was higher in buffaloes (66%) than cattle (53%). Besides, month-wise prevalence was higher in hot and humid months in both species. *Staphylococci* spp. (34%) were the most predominant bacterial isolates from mastitic milk, followed by *Escherichia coli* (19.4%), *Streptococci* spp. (9%), and *Klebsiella* spp. (8%). Most of the bacteria were susceptible to gentamicin (92%) and enrofloxacin (88%), when a panel of 16 different antimicrobials was tested. Nevertheless, most of the isolates were resistant to sulphamethoxazole (99%), lincomycin (98%), oxytetracycline (89%), ampicillin (86%), and doxycycline (85%). This study concludes a high prevalence of mastitis caused by *Staphylococcal* spp. in cattle and buffaloes belonging to the northwest of Pakistan, and gentamicin and enrofloxacin might be appropriate antimicrobial agents in the treatment of bovine mastitis.

## Introduction

Mastitis (inflammation of the mammary gland) is one of the most prevalent diseases of dairy animals (1). It is the most costly and devastating disease to the dairy industry because it imparts vast economic losses, compromising the health and welfare of animals, and due to its adverse effects on the quality and quantity of milk (1). Cattle and buffaloes (*Bubalus bubalis*) are the major dairy animals in Pakistan. The disease is associated with considerable alterations in milk chemical composition with a significant decrease in milk synthesis and changes in cell permeability (2). Bacterial pathogens are majorly (70%) involved in the etiology of mastitis. Besides, minor causes (30%) include non-infectious agents such as physical trauma, mechanical injuries to the gland, etc. (3). More than 135 types of bacterial species have been reported to be associated with bovine mastitis, but 20 different pathogenic bacteria are most commonly involved in mastitis of dairy animals (3, 4). The most common mastitis-causing bacterial pathogens are *Staphylococcus aureus, Escherichia coli, coagulase-negative staphylococci* (CNS), *Streptococcus dysgalactiae, Streptococcus uberis*, and *Streptococcus agalactiae* (4–6).

Generally, antimicrobial agents are used to treating bacterial infections including intramammary infections (IMI) in livestock. In dairy animals, mastitis and reproductive disorders are some of the most important reasons for frequent and prolonged use of antimicrobials (7). Quite usual, the treatment of mastitis takes a longer duration due to relapse and failure to flush out those bacteria which are resistant to a wide range of antimicrobials. The therapy of mastitis should be as efficient and quick as possible; however, there is a rapid increase in the prevalence of multidrug-resistant bacteria globally (5). According to the World Health Organization (WHO), an efficient monitoring system for the antimicrobial resistance of various bacterial pathogens is urgently required at national levels to fulfill the requirements of international standards (8). Data regarding the prevalence of bovine mastitis and the knowledge of pathogens causing mastitis is important to chalk out prevention and control strategies and to adopt appropriate therapeutic protocols (4).

In Pakistan, national surveillance programs for monitoring the prevalence of bovine mastitis and antimicrobial resistance in bacterial pathogens are lacking. Only some discrete studies were conducted in different areas of Pakistan (9, 10). Thus, there is always a dire need to monitor the prevalence of mastitis at local levels, as well as its etiology, and to evaluate antimicrobial susceptibility of bacterial isolates. Therefore, this study was carried out with the objectives to investigate: (i) the prevalence of bovine mastitis in cattle and buffaloes located in the northwest of Pakistan; (ii) detection of bacterial etiology of mastitis; and (iii) antimicrobial susceptibility of the bacterial isolates.

## Materials and Methods

### Statement of Ethics

This cross-sectional prevalence study was performed in accordance with ethical guidelines of Veterinary Research Institute Peshawar, Khyber Pakhtunkhwa.

### Sample Collection and Processing

Milk samples were aseptically collected by local dairy farmers within our connection according to standard procedures. Dairy farmers were properly guided for collection of milk samples from their cattle and buffaloes (Bubalus bubalis). Milk samples (*n* = 1,566) were collected from cattle (*n* = 1,096) and buffaloes (*n* = 470) by their owners or by local veterinarians and processed for the detection of mastitis at the Mastitis section, Center of Microbiology and Biotechnology, Veterinary Research Institute Peshawar, Khyber Pakhtunkhwa, Pakistan, from July 2018 to June 2019. Milk samples were mostly brought by smallholding dairy farmers having one to three animals. According to data provided by the farm owners, most of the farms were semi-Paka, made of sand and bricks, or Kacha, made of sand and mud. Farmers were guided to collect aseptical milk samples according to the standard protocols of the National Mastitis Council. They were advised to collect composite milk samples in duplicate per animal. Dairy milk samples were brought by farmers from their dairy cattle (*n* = 1,096) and buffaloes (*n* = 470), belonging to different areas of Khyber Pakhtunkhwa province including districts Peshawar (*n* = 744), Charsada (*n* = 125), Nowshera (*n* = 57), Khyber (*n* = 67), Swabi (*n* = 38), and other areas (*n* = 65). The sample size from different areas was proportional to the local total number of cattle and buffaloes.

### Clinical and Subclinical Mastitis Detection

Dairy farmers were guided to recognize clinical mastitis from the clinical signs and abnormal changes in milk (10). Subclinical mastitis was tested using the California Mastitis Test (CMT), according to the recommendations of commercially available CMT Kit (Techni. Vet., Inc. USA). In addition, the scoring system of CMT was considered (scoring at +, ++, and +++, corresponding to mildly positive, moderately positive, and strongly positive, respectively), indicating the intensity of subclinical mastitis in cattle and buffaloes. Month-wise and area-wise prevalence of mastitis in cattle and buffaloes were also enumerated.

### Identification of Bacterial Isolates Associated With Mastitis

A total of 633 mastitic milk samples were further processed for the identification of bacteria at the Bacteriology section of the Center of Microbiology and Biotechnology. Bacteria were identified up to the genus levels as described previously (11). Standard protocols were adopted to carry out bacteriology of milk samples according to the guidelines of the National Mastitis Council (NMC, http://www.nmconline.org/wp-content/uploads/2016/09/Procedures-for-CollectingMilk-Samples.pdf and University of MN Laboratory for Udder Health, https://www.vdl.umn.edu/sites/vdl.umn.edu/files/sample-collection-guide-withpictures.pdf). Additionally, presumptively identified *E. coli* isolates were further confirmed using molecular assay by thermal cycler (Bio-Rad T100™) using forward primers (5′-TGG TAA TTA CCG ACG AAA ACG GC-3′) and reverse primers (5′-ACG CGT GGT TAC AGT CTT GCG-3′), targeting *uidA* at 62°C annealing temperature as described by Tantawiwat et al. (12).

### Antimicrobial Susceptibility Testing

Antimicrobial susceptibility testing of the bacterial isolates from cases of clinical and subclinical mastitis was performed on Mueller-Hinton agar (Difco™) using the Kirby-Bauer disk diffusion method according to the Clinical Laboratory Standard Institute (13). A panel of 16 different commercially available discs of antimicrobial agents (Oxoid™, Thermo Fisher Scientific Inc., Carlsbad, CA, USA) was tested. The antimicrobial agents included ampicillin, amoxicillin, amoxicillin + clavulanate, cefotaxime + clavulanate, oxytetracycline, doxycycline, gentamicin, kanamycin, lincomycin, streptomycin, erythromycin, norfloxacin, enrofloxacin ciprofloxacin, chloramphenicol, and sulphamethoxazole.

### Statistical Analysis

The data were analyzed by STATA software using descriptive analysis. Chisq test was applied to compare the prevalence and severity of clinical mastitis between samples from cattle and buffaloes.

## Results

### Prevalence of Mastitis in Cattle and Buffaloes

The overall prevalence of clinical and subclinical mastitis was 17 and 57% in milk samples from cattle and buffaloes belonging to various areas of Khyber Pakhtunkhwa province (Table 1). The highest prevalence of clinical mastitis (20%) was noted in cattle, whereas subclinical mastitis was 53% in cattle belonging to various breeds, mostly crossbred Holstein Frisian and Jersey breeds. However, the prevalence of subclinical (66%) was highest in buffaloes, while clinical mastitis was 11%. Blood mixed milk samples were detected in 4.7% (*n* = 51) and 4% (*n* = 19) cows and buffaloes, respectively (Table 1). The prevalence of clinical mastitis in cattle was significantly higher than that in buffaloes (*p* < 0.5), while the prevalence of subclinical mastitis in cattle was significantly lower than that in buffaloes (*p* < 0.5).

**Table 1:** The prevalence of bovine mastitis in cattle and buffaloes.

**Species**	**Samples processed**	**Clinical mastitis**		**Subclinical mastitis**		**Blood in milk**	
		***n***	**%**	***n***	**%**	***n***	**%**
Cattle	1,096	220	20.1	582	53.1	51	4.7
Buffaloes	470	51	10.9	312	66.4	19	4.0
Total	1,566	271	17.3	894	57.1	70	4.5

The results also revealed that strongly positive intensity (+++) was noted in 64% cases of subclinical mastitis, followed by moderate intensity (++; 17%) and mild intensity (+; 17%) in cattle and buffaloes using California Mastitis Test. The higher prevalence of strongly positive subclinical mastitis (+++) was noted in cattle (69%), compared to buffaloes (55%), as shown in Table 2. The prevalence of strongly positive subclinical mastitis in cattle was significantly higher than that of buffaloes (*p* < 0.5).

**Table 2:** The severity of subclinical mastitis in cow and buffaloes.

**Species**	**Samples processed**	**Traces**		**Mild**		**Moderate**		**Severe**	
		***n***	**%**	***n***	**%**	***n***	**%**	***n***	**%**
Cattle	582	08	1.4	84	14.4	91	15.6	399	68.6
Buffaloes	312	14	4.5	65	20.8	63	20.2	170	54.5
Total	894	22	2.5	149	16.7	154	17.2	569	63.7

### Month-Wise and Area-Wise Prevalence of Mastitis

Month-wise prevalence of bovine mastitis in cattle and buffaloes of Khyber Pakhtunkhwa is shown in Table 3. Highest prevalence of mastitis was noted in the hot and humid months of July, August, and September in cattle and buffaloes. However, the lowest prevalence of mastitis in cattle was observed in May, but in buffalos, the prevalence of mastitis was lowest in the month of November (Table 3). The area-wise highest prevalence of mastitis was observed in cattle (76.9%) and buffaloes (80.2%) of district Peshawar, followed by the Sawabi and Nowshera districts (Table 4).

**Table 3:** Month-wise prevalence of bovine mastitis in cattle and buffaloes.

**Months**	**Cow**			**Buffaloes**		
	**Samples**	***n***	**%**	**Samples**	***n***	**%**
January	75	47	62.7	87	67	77.0
February	57	47	82.5	41	33	80.5
March	60	45	75.0	39	27	69.2
April	106	85	80.2	45	38	84.4
May	122	56	45.9	26	23	88.5
June	73	51	69.9	8	7	87.5
July	65	55	84.6	09	8	88.9
August	168	120	71.4	36	33	91.7
September	134	114	85.1	27	23	85.2
October	72	55	76.4	54	40	74.1
November	95	74	77.9	53	26	49.1
December	69	53	76.8	45	38	84.4

**Table 4:** Area-wise prevalence of bovine mastitis in cattle and buffaloes.

**Areas**	**Cow**			**Buffaloes**		
	***n***	**Positive**	**%**	***n***	**Positive**	**%**
Peshawar	744	572	76.9	358	287	80.2
Charsada	125	82	65.6	21	12	57.1
Nowshera	57	37	64.9	69	52	75.4
Khyber	67	42	62.7	03	02	67.0
Sawabi	38	29	76.0	15	08	53.3
Other areas^*^	65	40	61.5	04	02	50.0

* *Other areas included scattered samples from Kohat, Karak, Bannu, Hazara, Dara Adam Kheil, etc*.

### Bacteriology of Mastitic Milk Samples

Table 5 shows the prevalence of different bacterial isolates from mastitis-positive cases of cattle and buffaloes of Khyber Pakhtunkhwa. The results showed the most common isolated bacteria were *Staphylococci* spp. (34%), followed by *Escherichia coli* (19%), *Streptococci* spp. (9%), and *Klebsiella* spp. (8%). Minor bacteria recovered from mastitic milk samples were *Salmonella* spp. (2%), *Proteus* spp. (1%), and *Candida* spp. (0.6%). However, no growth was observed in 16% of mastitic milk samples. A total of 186 (19%) *E. coli* isolates were confirmed by PCR amplification of *uidA* gene with amplicon size of 147 bp.

**Table 5:** The bacteriology of milk (*n* = 633) of cattle and buffaloes suffering from mastitis.

**Bacteria isolated**	**Numbers (*n*)**	**Percentage (%)**
*Staphylococci* spp.	215	34.0
*Escherichia coli*	186	19.4
*Streptococcus* spp.	58	09.2
*Klebsiella* spp.	50	07.9
*Salmonella* spp.	14	02.2
*Proteus* spp.	07	01.1
*Candida* spp.	04	00.6
Culture negative	99	15.6

### Antimicrobial Susceptibility Testing

The results of antimicrobial susceptibility testing of the bacterial isolates are shown in Table 6. Gentamicin, enrofloxacin, and ciprofloxacin showed the highest susceptibility to various bacterial isolates from milk of cows and buffaloes suffering mastitis. Overall, 92% of the bacterial isolates were susceptible to gentamicin, followed by 88% susceptibility to enrofloxacin and 79% to ciprofloxacin. On the contrary, the highest resistance was noted against sulphamethoxazole (99%), followed by lincomycin (98%), oxytetracycline (89%), ampicillin (86%), and doxycycline (85%).

**Table 6:** Antimicrobial susceptibility of bacterial isolates from mastitic cattle and buffaloes.

**Antimicrobial agents**	**Concentration (μg)**	**Susceptible isolates**		**Resistant isolates**	
		***n***	**%**	***n***	**%**
Ampicillin	10	35	13.9	217	86.1
Amoxicillin	10	68	34.0	132	66.0
Amoxicillin/clavulanate	20/10	66	38.4	106	61.6
Cefotaxime/clavulanate	30/10	35	14.6	205	85.4
Oxytetracycline	30	26	10.7	216	89.3
Doxycycline	30	34	16.8	168	83.2
Gentamicin	10	205	91.5	19	08.5
Kanamycin	30	92	52.0	85	48.0
Lincomycin	10	4	02.0	204	98.0
Streptomycin	10	38	32.8	78	67.2
Erythromycin	15	50	22.3	174	77.7
Norfloxacin	10	149	65.4	79	34.6
Enrofloxacin	10	233	88.3	31	11.7
Ciprofloxacin	05	192	79.3	50	20.7
Chloramphenicol	30	212	84.1	40	15.9
Sulphamethoxazole	23.75	2	00.7	291	99.3

## Discussion

Mastitis is one of the most prevalent diseases of dairy animals all over the world (1), including Pakistan (11, 14, 15). In the current study, 20 and 53% prevalence of clinical and subclinical mastitis was noted in cattle, whereas it was 11 and 66% in buffaloes belonging to different areas of Khyber Pakhtunkhwa. This prevalence of subclinical mastitis was quite higher than the previous reports of 35% subclinical mastitis in cattle of district Muzaffar Gharr (14), 30% in cattle of district Lahore (9), and 44% in Punjab (11), and our previously reported 42% subclinical mastitis in buffaloes of district D. I. Khan (10). This considerable increase in the prevalence of bovine mastitis is alarming, which might be because dairy farming is rapidly growing in Khyber Pakhtunkhwa, and it is reported that mastitis is significantly increasing with an increase in the number of dairy animals (10). The other reasons might be that the number of milk samples processed and areas under study in the present study were considerably more than the other studies. The prevalence of clinical mastitis was in line with previous studies which reported 11.5% clinical mastitis in cattle of the Jammu and Kashmir region (15) and 11% in Nili Ravi buffaloes (10). However, Mustafa et al. (9) and Sharif et al. (16) reported a higher prevalence of clinical mastitis in cattle and buffaloes. They reported a higher prevalence of 40% and 61% of clinical mastitis in buffaloes and cattle. The highest prevalence of clinical mastitis was observed in cattle followed by buffaloes, which is also in line with a previous study (9). This might be attributed to the decreased immunity of exotic and crossbred cattle in Pakistan. However, as buffaloes are local to the environment, they are more resistant to various diseases including mastitis. In addition, 4.7 and 4.0% blood-mixed milk samples were observed in cows and buffaloes, which is in agreement with other studies conducted in this region (9, 16). The prevalence of bovine mastitis in cattle was also in partial agreement with several global studies conducted in India (17), China (4), Ethiopia (18), and Poland (19). Additionally, the highest prevalence of mastitis was noted in the hot and humid months of summer in both species. Similar findings were also observed by Sinha et al. (17); they reported the highest incidence of mastitis during the monsoon season. The prevalence of mastitis varied with different regions, the highest prevalence being noted in the cattle (81%) and buffaloes (82%) of district Peshawar. Previous studies also reported that the prevalence of mastitis varied with geography (10, 11).

Bacteriology of mastitic milk samples from cattle and buffaloes showed that *Staphylococci* spp. (34%) and *E. coli* (19%) were the most frequently isolated bacterial pathogen from cattle and buffaloes suffering mastitis. This was followed by *streptococci* spp. (9%)*, Klebsiella* spp. (8%), *Salmonella* spp. (2%), and *Proteus* spp. (1%). This was in agreement with the other studies conducted in various regions of Pakistan (9, 11, 15, 20, 21), which also predominantly isolated *Staphylococci* spp. and *E. coli* from cases of bovine mastitis. We identified *E. coli* isolates (*n* = 186) by molecular assay using PCR amplification of *uidA* gene. This was in agreement with our previous work (5) and the study of Tantawiwat et al. (12). In a recent large-scale Chinese study, Song et al. (22) also reported that staphylococci spp. were the main pathogens associated with mastitis in cattle. Similarly, Bhat et al. (15) reported that *Staphylococcus aureus* (61%) was the most prevalent bacteria isolated from the mastitic cattle of Jammu and Kashmir, followed by *E. coli* (13%), coagulase-negative staphylococci (13.04%), *Streptococcus uberis* (4.35%), and *Streptococcus dysgalactiae* (8.69%). Gao et al. (4) and Ali et al. (5) also reported these pathogens from Chinese cattle. However, in another large-scale Chinese study by Gao et al. (4), they concluded that *E. coli* (14%) were the most prevalent bacteria isolated cases of bovine clinical mastitis followed by *Klebsiella* spp. (13%), coagulase-negative staphylococci (10%), *Streptococcus dysgalactiae* (11%), *Staphylococcus aureus* (10%), *Streptococcus agalactiae* (3%), and *Streptococcus uberis* (2%). This might be because the bacteria causing mastitis are changing with topographical and management conditions.

In the current study, gentamicin (92%), enrofloxacin (88%), and ciprofloxacin (79%) showed the highest susceptibility to various bacterial isolates from milk of cows and buffaloes suffering mastitis. However, the highest resistance of bacterial isolates was observed against sulphamethoxazole (99%), lincomycin (98%), oxytetracycline (89%), ampicillin (86%), and doxycycline (85%). This was in partial agreement with the study of Iqbal et al. (20); they also reported that gentamicin, enrofloxacin, norfloxacin, and kanamycin were the most effective antimicrobial drugs against the isolated bacteria from bovine mastitis (20). Similarly, several other studies also reported the highest susceptibility of bacterial isolates toward aminoglycosides including gentamicin, fluoroquinolone like enrofloxacin, and ciprofloxacin (11, 16). Notably, gentamicin and enrofloxacin antibiotics are listed in the approved list of antimicrobial agents approved for veterinary medicine by the World Health Organization and World Organization for Animal Health (23). This study, describes for the first time the prevalence of clinical and subclinical mastitis in a large number of dairy cattle and buffaloes belonging to different areas of Khyber Pakhtunkhwa, and identifies different bacterial pathogens associated with mastitis and their antimicrobial susceptibility profiles. Thus, this study would be beneficial for scientists, researchers, and clinicians working with the dairy sector to sketch prevention and control strategies and to adopt appropriate therapeutic protocols for bovine mastitis.

## Conclusions

This study concludes the high prevalence of clinical and subclinical mastitis in cattle and buffaloes belonging to the northwest of Pakistan. *Staphylococci* spp. was the major bacterial pathogen associated with mastitis. This indicates unhygienic and poor management practices at the local farms. In addition, based on susceptible antimicrobial profiling of the isolated bacterial pathogens from cases of bovine mastitis, gentamicin and enrofloxacin might be appropriate antimicrobial agents in the treatment of bovine mastitis.

## Data Availability Statement

The original contributions presented in the study are included in the article/supplementary material, further inquiries can be directed to the corresponding author/s.

## Ethics Statement

The animal study was reviewed and approved by Ethical Committee of Veterinary Research Institute Peshawar, Khyber Pakhtunkhwa. Written informed consent was obtained from the owners for the participation of their animals in this study.

## Author Contributions

TA, AR, MA, and GL designed and conceived the study. TA, Kamran, IW, and RU performed the experiments. TA, PS, and BH analyzed the data and wrote the article. BH and GL critically reviewed and revised the manuscript. All authors read and approved the final version.

## Funding

This study was carried out with the financial support of the Annual Development Program (ADP) of the Government of Khyber Pakhtunkhwa under ADP scheme No. 25/150090, with the title Establishment of Livestock Research and Development Station at Swabi.

## Conflict of Interest

The authors declare that the research was conducted in the absence of any commercial or financial relationships that could be construed as a potential conflict of interest.

## Publisher's Note

All claims expressed in this article are solely those of the authors and do not necessarily represent those of their affiliated organizations, or those of the publisher, the editors and the reviewers. Any product that may be evaluated in this article, or claim that may be made by its manufacturer, is not guaranteed or endorsed by the publisher.
